# “Feel of the reservoir bag” ... A dying skill in midst of sophisticated equipment!

**DOI:** 10.4103/0019-5049.63627

**Published:** 2010

**Authors:** Rajeev Sharma

**Affiliations:** Department of Anaesthesia, ESI Hospital, Rohini, New Delhi, India

Sir,

I read with interest the article on Severe intraoperative brain bulge due to endotracheal tube obstruction by a thick mucous plug by Singhal *et al*.[1] I congratulate the authors for averting a near-fatal situation using timely change of the blocked endotracheal tube. There was a rise in EtCO_2_ levels and gradually increasing airway pressures in the child being ventilated with the help of a ventilator.

I think the problem could have been detected earlier had they been ventilating the lungs manually. The hands of an experienced anaesthetist are trained to instantaneously detect a change in the compliance of the reservoir bag provided the “initial feel” of the bag is used as a base line. Significant time must have elapsed before the EtCO_2_ rose to 60 mm Hg. Unfortunately, with the advent of sophisticated equipment and monitors; much less emphasis is placed nowadays on the 'feel' of the bag.

The steps of management of the presented case could have been better sequenced. When the airway pressures and the EtCO_2_ levels were rising, a quick check by manual ventilation and feel of compliance followed by ruling out endotracheal tube obstruction (by suctioning) or circuit obstruction and bronchospasm before giving supplemental doses of opioids and muscle relaxants and performing an arterial blood gas analysis, could have avoided the severe intraoperative brain bulge that was seen in the presented case.

## References

[1] Singhal V, Pandia MP, Dash H (2009). Severe intraoperative brain bulge due to endotracheal tube obstruction by a thick mucous plug. Indian J Anesth.

